# Estimating Transmission Potential of H5N1 Viruses Among Humans in Egypt Using Phylogeny, Genetic Distance and Sampling Time Interval

**DOI:** 10.3389/fmicb.2019.02765

**Published:** 2019-12-03

**Authors:** Wessam Mohamed, Kimihito Ito, Ryosuke Omori

**Affiliations:** Division of Bioinformatics, Research Center for Zoonosis Control, Hokkaido University, Sapporo, Japan

**Keywords:** avian influenza, Egypt, H5N1, human-to-human transmission, *R*_0_, statistical model

## Abstract

In 2014 and 2015, the number of human cases of H5N1 avian influenza virus infections had increased dramatically in Egypt. This increase might be related to increase in the transmission potential of the virus among humans. To clarify the cause of the increase in H5N1 human cases, we investigate the transmissibility of H5N1 viruses among humans via estimating the basic reproduction number *R*_0_ using nucleotide sequences and sampling dates of viruses. To this end, full-length hemagglutinin gene sequences of human and avian H5N1 influenza viruses isolated from 2006 to 2016 in Egypt were obtained from the NCBI influenza virus resource. Taking into account the phylogeny, genetic distance, sampling time difference among viruses, *R*_0_ was estimated to be 0.05 (95% CI: 0.01, 0.13) assuming that human-to-human transmissions occurred within a city, 0.23(95% CI: 0.14, 0.35) assuming human-to-human transmissions among cities. Our results indicate that human-to-human transmission of H5N1 viruses in Egypt is limited, and the large increase in human cases is likely attributed to other factor than increase in human-to-human transmission potential.

## Introduction

The H5N1 avian influenza virus is of great concern to the public health in the world. According to the World Health Organization (WHO), more than 860 human cases have been clinically diagnosed as H5N1 infections in 17 countries by the 24th of June 2019 (World Health Organization [WHO], 2019). In 2009, the World Organisation for Animal Health (OIE) reported that H5N1 viruses became enzootic in poultry populations in Bangladesh, Cambodia, China, Egypt, India, Indonesia, Laos, Nepal and Vietnam (Tarantola et al., 2010). H5N1 viruses have occasionally transmitted to humans and caused severe respiratory disorders leading to death (Yuen et al., 1998). Previous studies have shown that H5N1 can acquire the ability to transmit in ferrets (Herfst et al., 2012; Imai et al., 2012). Although the human-to-human transmission of the virus is limited, family case clusters of H5N1 infections were reported from Sumatra (Yang et al., 2007), Cambodia (Chea et al., 2014), Thailand (Ungchusak et al., 2005), China (Wang et al., 2008), and Pakistan (World Health Organization [WHO], 2008).

H5N1 influenza viruses were first identified in Egypt among poultry in 2006 (Aly et al., 2008) and declared to be enzootic in 2008 (Kayali et al., 2016). The number of human cases in Egypt has been increasing dramatically since 2014 (Refaey et al., 2015). Two-thirds of the human cases reported in the world by 2019 were from Egypt (World Health Organization [WHO], 2019). The increase in 2014 might be either related to increase in the transmission potential of the H5N1 virus among human populations or other factors. Furthermore, Arafa A. S. et al. (2016) found that some H5N1 viruses isolated in Egypt during 2014–2015 had the ability to be transmitted via respiratory droplets between ferrets, although the transmission efficiency of these H5N1 viruses in ferrets may be very low. However, in the slight possibility that H5N1 viruses may acquire airborne transmission in a natural environment, the identification of the transmission potential of H5N1 viruses in humans is crucial to improve the control measures that can be used to contain virus spread in Egypt.

One way to estimate the transmissibility of infectious diseases is by measuring the basic reproduction number (*R*_0_), which is defined as the average number of secondary cases originating from a primary infected case in a whole susceptible population (Vynnycky, 2010). To estimate *R*_0_ of avian virus infections in a human population, the number of human cases and/or cases with history of bird contact are frequently used (Nishiura et al., 2013). However, the data on contact with poultry may not be accurate, because data are biased by the recall bias or missing data. Lack of epidemiological data is an obstacle for the estimation of *R*_0_ of avian virus infections in a human population.

Recently, various types of epidemiological information have been used to estimate *R*_0_. Chong et al. used travel data, i.e., arrival times of infected cases in different countries (Chong et al., 2017). Farrington et al. used age-stratified serological survey data to estimate *R*_0_ of hepatitis A, mumps, rubella, parvovirus, *Haemophilus influenzae*, and measles infection (Farrington and Kanaan, 2001). On the other hand, Pybus et al. (2001) developed a method to estimate *R*_0_ of an infectious disease using nucleotide sequences of pathogens, showing nucleotide sequences have the ability to infer the magnitude of infectious diseases transmission.

In this study, we assess the transmission potential of H5N1 viruses among humans in Egypt. We measure transmission potential by *R*_0_ estimated from nucleotide sequences, sampling time, and sampling location of H5N1 viruses isolated in Egypt during 2006–2016.

## Materials and Methods

### Sequence Data

Nucleotide sequences of the hemagglutinin (HA) gene of H5N1 influenza viruses isolated from humans and birds in Egypt were downloaded from the NCBI Influenza Virus Database (Bao et al., 2008). We selected full-length and nearly full-length HA sequences in the database, and nucleotide sequences of HA of 73 human isolates and 531 avian isolates from 2006 to 2016 were obtained. For each nucleotide sequence we recorded the city and the date where and when its virus was sampled. Thirteen human sequences that lacked sampling date information were excluded from subsequent analyses. The GenBank accession numbers, strain names, sampling dates, and sampling locations of nucleotide sequences used in this study can be found in the Supplementary Table S1. For the clustering analysis described below, *p*-distance (Nei and Kumar, 2000) among all pairs of sequences were calculated. For each city where viruses were sampled, the latitude and longitude of the city were obtained from Egypt Cities Database in Simplemaps.Com (2019). Then, geographical distances between cities were calculated using the “geosphere” library in R software.

### Phylogenetic Analysis

To visualize evolutionary relationships among Egyptian H5N1 viruses isolated from birds and humans, a phylogenetic tree was constructed using HA sequences of avian and human isolates. We used the maximum composite likelihood with general time reversible substitution model in MEGA software version 6.06 (Tamura et al., 2013) to construct the phylogenetic tree. The genetic distance between two viruses are depicted to be proportional to the summation of horizontal distances of branches connecting them. Clade information of H5N1 viruses are shown using different colors. Human viruses are emphasized by marks.

### Detection of Human Case Clusters in Phylogenetic Tree

To find possible human-to-human transmissions, we first identified clusters of human isolates in the phylogenetic tree. Clusters of human isolates were identified as follows. Each human isolate is considered to belong to a cluster. There may be a cluster with a single virus, and we call such a cluster a singleton cluster. Two human isolates are considered to belong to the same cluster if they are connecting with one or two internal nodes in the phylogenetic tree. Applying this criterion for all pairs of human isolates, we get clusters of human isolates.

### Estimation of Human-to-Human Transmission Potential

To estimate the number of human-to-human transmission events in an infection chain, we employ a mathematical model describing human-to-human transmission. We hypothesized all human index cases of H5N1 viruses are avian-to-human transmission. Let *n* be the number of avian-to-human transmissions and *S* be the total number of human cases. For an infectious chain *i*, which starts from an individual infected from a bird and ends with a person who has not transmitted virus to another person, let *x*_*i*_ denote the number of individuals in the infectious chain *i*. We assume the probability of observing the “*i*”-th chains with length *x*_*i*_ follows a geometric distribution with the “success” probability *p*, which is considered as the probability of a human-to-human transmission. The likelihood function *L* of human-to-human transmission probability *p* can be written as follow:

$$L\,\left(p;x_{1},\ldots ,x_{n}\right)=\prod_{i=1}^{n}p^{x_{i}-1}\,\left(1-p\right).$$

Since, the summation of *x*_*i*_ over *i* is equal to the total number of human cases *S*, the maximum likelihood estimate of *p* is given by,

$$\hat{p}=\frac{\sum_{i=1}^{n}x_{i}-n}{\sum_{i=1}^{n}x_{i}}=1-\frac{n}{S}.$$

*R*_0_ is the expected number of secondary cases from single case in the entire susceptible population. Assuming that *R*_0_ is smaller than or equal to 1, the representative value for the estimate of *R*_0_ can be given by,

$$R_{0}=\hat{p}=1-\frac{n}{S}.$$

**FIGURE 1:**
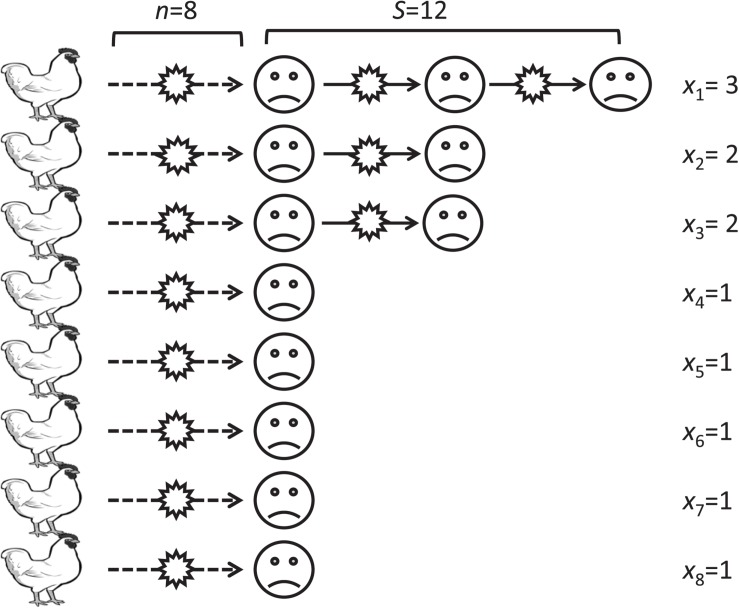
An illustrative example of infection chains in our model. A virus transmits from a bird to a human (broken lines) then transmits from a human to another human and so on (solid lines). The *x*_*i*_ represent the length of the *i*th transmission chain.

Figure 1 illustrates an example of infection chains. In this example, the number of avian to human transmissions, *n*, is eight. The length of infection chains started from these avian-to-human transmissions are three, two, two, one, one, one, one, and one, and the total number of human cases, *S*, is 12. The *R*_0_ for this example is calculated to be 1–8/12 = 0.33.

We calculate the confidence interval (CI) for the *R*_0_ estimate using a likelihood ratio test. The critical value of *R*_0_ in our analysis is 1.0, because no major epidemic will occur when *R*_0_ is smaller than 1.0. Our method can applicable only for infections in sporadic outbreaks and cannot be used for infections in an endemic situation, since the length of transmission chain is assumed follow the geometric distribution. All the analysis was done using the statistical software R, and its R codes are available in Supplementary Data Sheet S1. The detailed process of statistical analysis is described in Supplementary Data Sheet S2.

### Clustering Analysis Using Genetic Distance, Sampling Time Interval, and Geographical Distance

**FIGURE 2:**
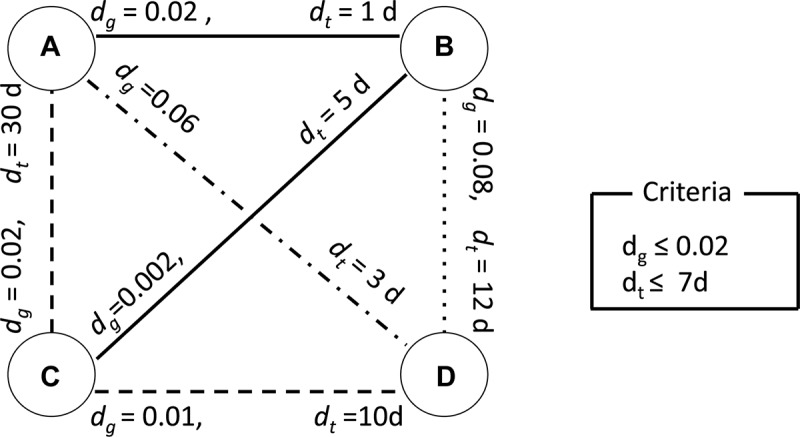
An illustrative example of clustering analysis using two criteria. The nodes A, B, C, and D represent viruses isolated from humans. The *d*_*g*_ on each edge connecting two nodes represents the genetic distance between corresponding viruses. The *d*_*t*_ on each edge represents the sampling time interval between the corresponding viruses. This example uses 0.02 as clustering threshold for genetic distance and 7 days for sampling time interval. A solid line represents a pair of viruses satisfying both criteria. A dashed line represents a pair of viruses satisfying only the criteria for genetic distance. A dash-dot line represents a pair of viruses satisfying only the criteria for sampling time interval. A dotted line represents a pair of viruses which does not satisfy with any of the criteria.

Possible human-to-human transmissions were identified using criteria of genetic distance, sampling time interval, and geographical distance. Figure 2 illustrates how our clustering algorithm works with genetic distance and sampling time interval. In this example, we have four viruses isolated from humans. The *d*_*g*_ on edges connecting two viruses represents the genetic distance between corresponding viruses. The *d*_*t*_ on edges represents the sampling time interval between the corresponding viruses. If we set genetic distance threshold to 0.02 and use only genetic distance as a criterion, then edges A–B, A–C, B–C, and C–D satisfy the criterion and A, B, C, and D are considered as a single cluster. The clustered sequences were sorted by their sampling time and transmission chains were reconstructed. We consider adjacent pairs in the transmission chain as candidate transmission pairs. In this case, we have 4 human cases and one transmission chain and *R*_0_ is calculated to be 1–1/4 = 0.75. If we set the sampling time interval threshold to 7 days and use sampling time as another criterion in addition to the genetic distance criterion, then A–B and B–C satisfy these two criteria, grouping A, B, and C to the same cluster and making D to be a singleton cluster. Then *R*_0_ is calculated to be 1–2/4 = 0.5. We can extend this to work with geographical distance in the same manner.

### Sensitivity Analysis

To assess the robustness of the *R*_0_ estimate, we conducted sensitivity analyses with respect to combinations of threshold values for the genetic distance, sampling time interval, and geographical distance. First, we varied genetic distance from 0 to 0.01 and calculated *R*_0_ and its 95% CIs. The 95% CI was calculated using the likelihood ratio test method. To obtain the distribution of sampling time interval and geographical distance between candidate transmission pairs, we used a fixed threshold for genetic distance between human viruses at a *p*-distance of 0.004488. This *p*-distance value is the between-households genetic variation, which is calculated by adding the mean value of between-households genetic distance and 1.96 times its standard deviation according to a previous study (Thai et al., 2014). Sequences in the clusters identified by phylogenetic analysis were also analyzed in the same manner.

To investigate the effect of sampling time interval threshold on the *R*_0_ estimate, we fixed the genetic distance threshold at the between-households genetic variation and varied the sampling time threshold from 0 to 60 days, which is a sufficient time for the infectious period of human influenza. For the sensitivity analysis using geographical distance, we fixed the genetic distance threshold at the between-households genetic variation and varied the geographical distance threshold from 0 to 700 km, which is the maximum distance within human clusters identified from genetic distance. We also conducted sensitivity analyses of sequences in the clusters identified by phylogenetic analysis in the same manner.

### Estimation of *R*_0_ From Phylogeny, Genetic Distance, Sampling Time Interval, and Geographical Distance

To obtain representative estimates of *R*_0_, we set threshold values of genetic distance, sampling time interval, and geographical distance. The threshold of genetic distance was set to 0.004488, which is the genetic distance observed in the between-household transmissions of H1N1 viruses (Thai et al., 2014). Threshold of sampling time interval was set to 30 days, which is the maximum value of the first cluster in the histogram of sampling time intervals. Since maximum duration of viral shedding is around 20 days (Loeb et al., 2012; Ng et al., 2016), the thresh hold of 30 days is an enough period to capture human-to-human transmissions. If it is assumed that human-to-human transmissions occurred only within household, we set the geographical distance threshold was 0 km (within a city), which is the geographical distance between the same city.

## Results

### Phylogenetic Analysis

**FIGURE 3:**
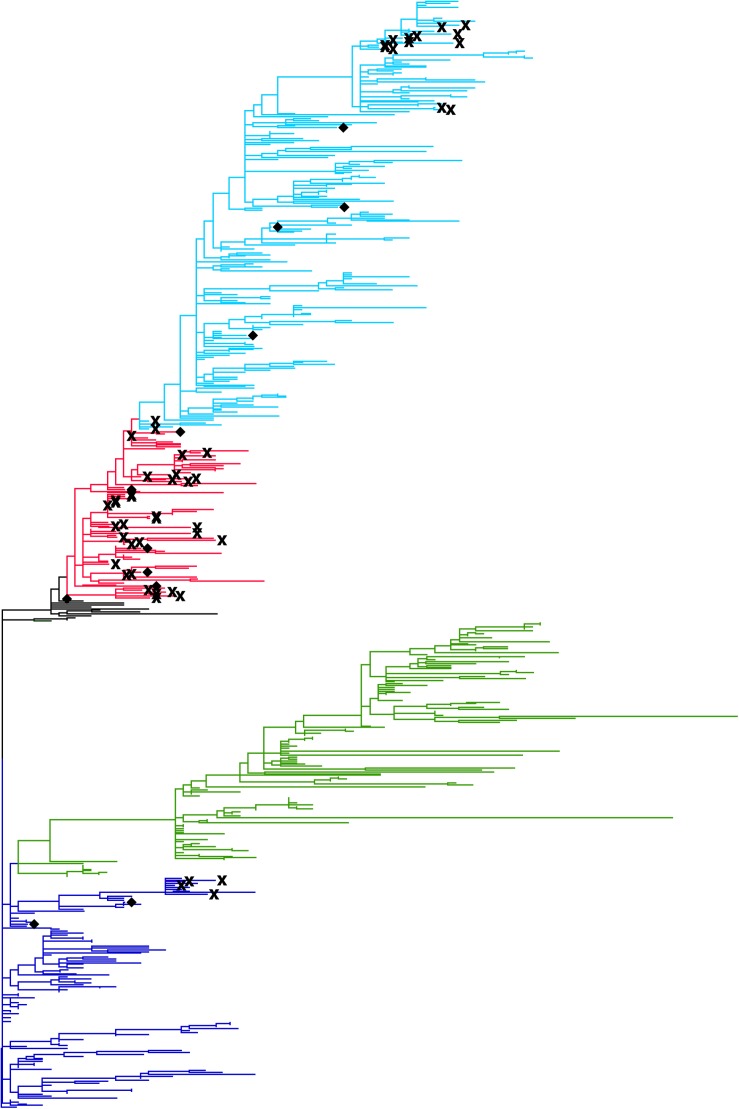
Phylogenetic tree of the HA gene of H5N1 avian influenza A viruses isolated in Egypt. The tree was constructed with the maximum composite likelihood with general time reversible substitution model in MEGA software version 6.06. Tips of the tree represent reported viral sequences. The genetic distance between two viruses are depicted to be proportional to the summation of horizontal distances of branches connecting them. Human viruses are emphasized by marks; human viruses identified in a human cluster containing more than one human isolate were represented by cross marks. Human viruses in a singleton cluster were represented by diamonds. Clade information of H5N1 viruses are shown using different colors; clade 2.2 are shown in blue, clade 2.2.1.1 in green, clade 2.2.1 in red, and clade 2.2.1.2 in light blue.

A phylogenetic tree was constructed from HA sequences of avian and human viruses isolated in Egypt from 2006 to 2016 (Figure 3). The phylogenetic tree has four major clades of clade 2.2, 2.2.1, 2.2.1.1, and 2.2.1.2 (Arafa A. et al., 2016). The 60 human sequences (cross and diamond marks) were distributed on subtrees of clade 2.2 (blue), 2.2.1 (red), and 2.2.1.2 (light blue) while clade 2.2.1.1 (green) did not contain human isolates, indicating that most avian-to-human transmissions were attributed to the avian viruses of clade 2.2, 2.2.1, and 2.2.1.2. From 60 sequences of human isolates, a total of 26 human clusters were found from connecting patterns of human sequences in the phylogenetic tree. Of these clusters, 12 were singleton clusters (diamonds) and 14 have more than one human sequence. These 14 clusters (cross marks) are candidates of possible human-to-human transmissions. The clade 2.2.1 has 9 candidate clusters of human-to-human transmissions, clade 2.2.1.2 has 4, and clade 2.2 has 1. Clade 2.2.1.1 has no candidate clusters for human-to-human transmissions. In total, 34 human-to-human transmission events were suggested from the phylogenetic tree. *R*_0_ was estimated to be 1–26/60 = 0.57 with its 95% CI from 0.44 to 0.69.

### Estimation of *R*_0_ From Genetic Distance

**FIGURE 4:**
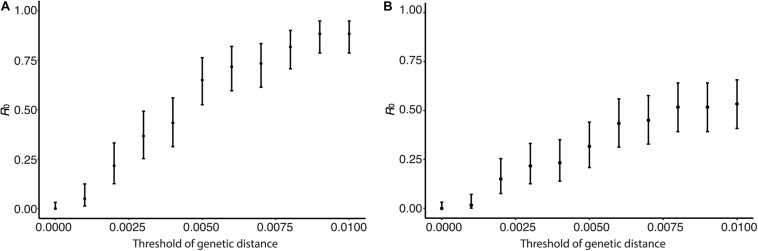
Effect of genetic distance threshold on the estimate of *R*_0_ using genetic distance. **(A)** Human viruses were clustered by genetic distances. **(B)** Human viruses were clustered by phylogeny and genetic distances.

Human isolates were clustered by genetic distances, and *R*_0_ and its CI were calculated from the clustering results. Figure 4 shows the sensitivity of *R*_0_ estimate with respect to genetic distance. In Figure 4A, clusters were identified using only genetic distances among human viruses without using the phylogenetic tree. *R*_0_ was estimated to be 0.00 with its 95% CI from 0.00 to 0.032 when the genetic distance threshold was 0.00. *R*_0_ increased as the genetic distance threshold increased, and *R*_0_ was estimated to be 0.883 with its 95% CI from 0.79 to 0.95 when the genetic distance threshold was 0.01. *R*_0_ was estimated to be 0.55 with its 95% CI from 0.42 to 0.67 when the genetic distance threshold was 0.004488, which is the between-household genetic variation. In Figure 4B, clusters were identified using phylogenetic tree of human and avian viruses and genetic distances among viruses clustered together. *R*_0_ was estimated to be 0.30 with its 95% CI from 0.19 to 0.42 when the genetic distance threshold was the between-household genetic variation. *R*_0_ was estimated to be 0.53 with its 95% CI from 0.41 to 0.66 when the genetic distance threshold was 0.01.

### Distribution of Sampling Time Interval and Geographical Distance Among Human Viruses

**FIGURE 5:**
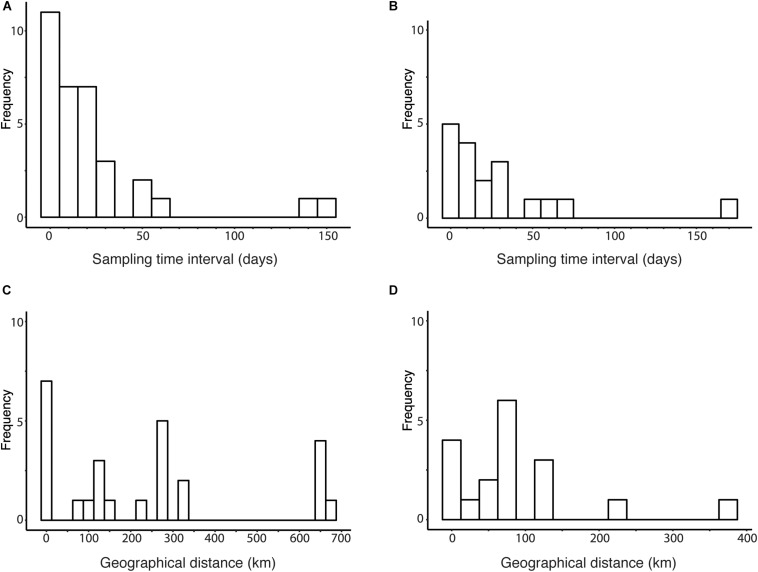
Distribution of sampling time interval and geographical distance among human viruses. **(A)** Histogram of sampling time intervals between human viruses clustered by genetic distances threshold at the between-household genetic variation. **(B)** Histogram of sampling time intervals between human viruses clustered by phylogeny and genetic distances threshold at the between-household genetic variation. **(C)** Histogram of geographical distances between human viruses clustered by genetic distances threshold at the between-household genetic variation. **(D)** Histogram of geographical distances between human viruses clustered by phylogeny and genetic distances threshold at the between-household genetic variation.

To determine the range of parameter values in the sensitivity analysis of *R*_0_ estimate, we analyzed the distribution of the parameters. The distribution of sampling time intervals and geographical distances among candidate transmission pairs were obtained by fixing the threshold for genetic distance between human viruses at the between-households genetic variation (Figure 5). Figure 5A shows histogram of time intervals among candidate transmission pairs in the cluster obtained using the between-households genetic distance among human sequences. Figure 5B uses phylogeny and the between-households genetic distance to obtain clusters of human sequences. The time intervals of candidate transmission pairs range from 0 to 149 days in clusters identified using genetic distance and from 1 to 170 days in clusters identified using phylogeny and genetic distance. Figure 5C shows histogram of geographical distances among candidate transmission pairs in the cluster obtained using the between-households genetic distance. Figure 5D uses phylogeny and the between-households genetic distance to obtain clusters of human sequences. The geographical distances of candidate transmission pairs ranges from 0 to 682.2 km in clusters identified using genetic distance and ranges from 0 to 383.3 km in clusters identified using phylogeny and genetic distance.

### Estimation of *R*_0_ From Genetic Distance and Sampling Time Interval

**FIGURE 6:**
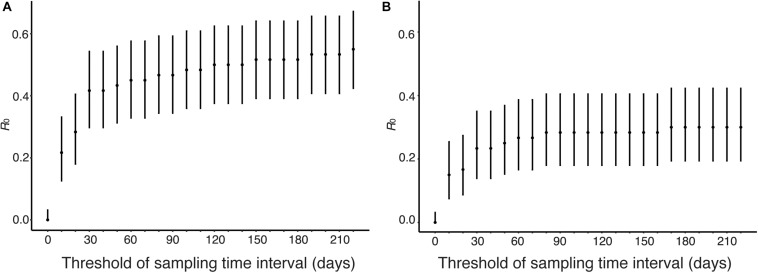
Estimation of *R*_0_ from genetic distance and sampling time interval. The effect of time interval threshold on the estimate of *R*_0_ is shown when genetic distance threshold fixed at the between-household variation. **(A)** Genetic distances and sampling time intervals were used to obtain candidate transmission pairs. **(B)** Phylogeny, genetic distances, and sampling time interval were used to obtain candidate transmission pairs.

Human viruses were clustered by genetic distances and sampling time interval, and *R*_0_ and its CI were calculated from the clustering results. Figure 6 shows sensitivity of *R*_0_ estimate with respect to sampling time interval threshold when genetic distance threshold was fixed at the between-household distance. Figure 6A uses genetic distances and sampling time intervals, and Figure 6B uses phylogeny and genetic distances and sampling time interval among human sequence clusters. *R*_0_ was estimated to be 0.55 (95% CI: 0.42, 0.67) without phylogeny and 0.30 (95% CI: 0.19, 0.42) with phylogeny when the sampling time interval threshold was 220 days, and these values decreased as sampling time interval threshold decreased. *R*_0_ was estimated to be 0.42 (95% CI: 0.30, 0.54) without phylogeny and 0.23 (95% CI: 0.14, 0.35) with phylogeny when the sampling time interval threshold was 30 days, which is the upper bound of sampling time interval between-household transmissions (Loeb et al., 2012; Ng et al., 2016).

### Estimation of *R*_0_ From Genetic Distance and Geographical Distance

**FIGURE 7:**
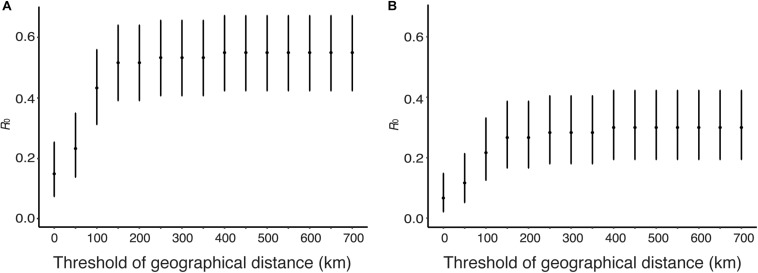
Estimation of *R*_0_ from genetic distance and geographical distance. The effect of geographical distance threshold on the estimate of *R*_0_ is shown when genetic distance threshold fixed at the between-household variation. **(A)** Genetic distances and geographical distance were used to obtain candidate transmission pairs. **(B)** Phylogeny, genetic distances, and geographical distance were used to obtain candidate transmission pairs.

Human viruses were clustered by genetic distances and geographical distances, and *R*_0_ and its CI were calculated from the clustering results. Figure 7 shows sensitivity of *R*_0_ estimate with respect to geographical distance threshold when genetic distance threshold was fixed at the between-household distance. Figure 7A uses genetic and geographical distances, and Figure 7B uses phylogeny and genetic distances and geographical distances among human sequence clusters. *R*_0_ was estimated to be 0.55 (95% CI: 0.42, 0.67) without phylogeny and 0.30 (95% CI: 0.19, 0.42) with phylogeny when the geographical distance threshold was 700 km, and these values decreased as geographical distance threshold decreased. *R*_0_ was estimated to be 0.15 (95% CI: 0.0751, 0.254) without phylogeny and 0.1 (95% CI: 0.041, 0.1923) with phylogeny when the geographical distance threshold was 0 km, which is the geographical distance between the same city.

### Estimation of *R*_0_ From Genetic Distance, Sampling Time Interval, and Geographical Distance

Using genetic distance threshold of 0.004488 and sampling time interval threshold of 30 days, human viruses were clustered by phylogeny, genetic distances, sampling time interval. Since, we do not have any restriction on the sampling location, this setting assumes that human-to-human transmissions can occur among different cities. In this setting, fourteen human-to-human transmissions were detected, and *R*_0_ was estimated to be 0.23(95% CI: 0.14, 0.35). If it is assumed that human-to-human transmissions occurred only within household and we set the geographical distance threshold was 0 km (within a city), which is the geographical distance between the same city, three human-to-human transmissions were detected. Based on this result, *R*_0_ was estimated to be 0.05 (95% CI: 0.01, 0.13).

Supplementary Table S2 shows the estimates of *R*_0_ for different combinations of genetic distance threshold, sampling time interval threshold, and geographical distance threshold. The upper bounds of 95% CI of our estimates of *R*_0_ using various thresholds were below 1.0.

## Discussion

In this paper, to elucidate the cause of the rapid increase in human cases of H5N1 influenza infections from 2014 to 2015 in Egypt, we have estimated the *R*_0_ of H5N1 infections in human population in Egypt using candidate transmission pairs identified from the nucleotide sequences, infection time, and geographic location of viruses. Sensitivity analysis shows that *R*_0_ is below unity, suggesting that major outbreak will not occur, with broad range of threshold values of genetic distance, sampling time interval, and geographical distance (Figures 4, 6, 7). Using phylogeny, genetic distance, sampling time interval, and geographical distance, we estimated *R*_0_ of 0.05 (95% CI: 0.01, 0.13) assuming that human-to-human transmissions occurred within a city, 0.23(95% CI: 0.14, 0.35) assuming human-to-human transmissions among cities, confirming previous studies that suggest human-to-human transmission of H5N1 viruses is rare in Egypt.

We have estimated the *R*_0_ of H5N1 infections in human population in Egypt using candidate transmission pairs identified from the nucleotide sequences, infection time, and geographic location of viruses. The nucleotide sequences of H5N1 influenza viruses were used to obtain candidate transmission pairs in two different ways. The first approach makes clusters of human viruses using genetic distances with respect to a given threshold value. This approach does not use nucleotide sequences of avian viruses. The second approach use a phylogenetic tree of influenza viruses constructed from nucleotide sequences of humans and birds and use genetic distance to divide clusters into transmission chains. The second approach reduces a possibility that two avian-to-human transmissions are clustered together. In fact, the estimated values of *R*_0_ were smaller when we analyzed sequences with phylogeny than without phylogeny (Figures 4A,B, 6A,B, 7A,B). There is a possibility that the *R*_0_ would further decrease if we had more avian sequences similar to human viruses. In sensitivity analysis, the effect of threshold values on *R*_0_ were smaller when with a phylogenetic tree (Figure 4B) than without a phylogenetic tree (Figure 4A).

Sensitivity analysis of sampling time interval showed a large effect on *R*_0_ between 0 days and 30 days (Figures 6A,B). *R*_0_ was estimated as 0.22, 0.28, and 0.42 when the sampling time interval threshold was 10, 20, and 30 days, respectively. It is known that the sampling time interval of transmission period is less than 30 days. Sensitivity analysis of geographical distance showed a large effect on *R*_0_ between 0and 150 km (Figures 7A,B). *R*_0_ was estimated as 0.067, 0.12, 0.22, 0.27 when the geographical distance threshold was 0, 50, 100, and 150 km, respectively. These results indicate that the threshold of sampling time interval and geographical distance are important variables to estimate *R*_0_ using our data. We estimated *R*_0_ with combinations of different threshold values for genetic distance, sampling time difference and spatial difference as sensitivity analyses. In all analyses, the upper bound of the 95% CI of *R*_0_ was below unity.

Although sensitivity of *R*_0_ against geographical distance is also high, the setting of geographical distance to detect clusters of human cases is not straightforward. Since several studies reported that most human-to-human transmissions of H5N1 occurred within their household (Ungchusak et al., 2005; Wang et al., 2008; Centers for Disease Control and Prevention, 2015), the threshold of geographical distance might be suitable to set 0 km, i.e., human-to-human transmissions occurred only within the same city. If this is the case, the *R*_0_ estimate is 0.05 (95% CI: 0.01, 0.13). However, the human cases of H5N1 in Egypt have not been well-understood whether they occur within or between household so far, and this assumption may underestimate *R*_0_. If we do not set any threshold of geographical distance, the *R*_0_ estimate is 0.23 (95% CI: 0.14, 0.35). However, this setting may overestimate *R*_0_. This setting assumes that genetic distance and sampling time difference are enough to determine the cluster of human cases formed by only human-to-human transmission. If multiple introductions of H5N1 viruses from avian to human occur, this setting may lead to misinterpret them as human-to-human transmission events. It is difficult to determine which of the two representative estimates of *R*_0_ is more reasonable because we cannot set the upper limit distance for human-to-human transmissions. To estimate an accurate *R*_0_, a detailed surveillance, e.g., contact tracing, is required. Regardless of the settings for threshold of geographical distance, the upper bound of the 95% CI of *R*_0_ was below unity.

Several studies have estimated *R*_0_ of H5N1 influenza viruses in human populations, most studies estimated *R*_0_ smaller than unity, ranges from 0.06 to 1.14 (Ferguson et al., 2004; Yang et al., 2007; Bettencourt and Ribeiro, 2008; Aditama et al., 2012; Saucedo et al., 2019). Our estimate of *R*_0_ assuming transmissions among cities, 0.23(95% CI: 0.14, 0.35), is comparable with most of estimates from these studies.

Human cases of the highly pathogenic avian influenza H5N1 had increased in 2014–2015, and three hypotheses for this increase can be raised; the increase in (i) human-to-human transmission potential, (ii) the susceptibility of humans to infection with these H5N1 viruses, (iii) avian-to-human transmissions. The upper bounds of 95% CI of our estimates of *R*_0_ using various methods were below unity, suggesting that major outbreaks due to human-to-human transmission are unlikely to occur in the current situation. Naguib et al. (2016) showed that human infections of H5N1 in Egypt in 2014–2015 are statistically linked to the increased outbreaks in poultry. This suggested that human cases were likely to be attributed to avian-to-human transmissions, although the increase in the susceptibility cannot be rejected. Furthermore, the distribution of geographical distance between human cases show unclear trend (Figures 5C,D), meanwhile, the distribution should be right-skewed if most human cases are attributed to human-to-human transmission. Sensitivity of *R*_0_ against geographical distance suggests that the outbreak of H5N1 among birds in Egypt occurs widely (diameter of the area is 200 km hypothesized from the saturation of *R*_0_ estimate in Figures 7A,B with assuming that human-to-human transmission is rare). The dramatic increase in human cases in Egypt would be attributed to the increase in the prevalence of H5N1 viruses among avian species, and avian-to-human transmissions in wide regions of Egypt may explain the unclear trend in the distribution of geographical distance between human cases.

This study proposed a method to estimate human-to-human transmissibility of zoonotic pathogens using nucleotide sequences as well as temporal and geographic information of infections. The integration of multiple types of data to the analyses can lead a more accurate estimation than analysis using a single type of data. However, there are some limitations in this study. First, we did not include exposure history to birds in our analysis. WHO reported 65 human cases from January 26 to March 3, 2015, of which 63 cases had exposure to poultry or poultry markets, and the exposure history of one case was still under investigation at the time of the report (World Health Organization [WHO], 2015a). From March 3 to 31, 2015, there were 37 human cases, of which all but one case had exposure to poultry or poultry markets, and the exposure history of the one case was still under investigation (World Health Organization [WHO], 2015b). The inclusion of bird contact information may change the estimates of *R*_0_. There is no link between the sequence data used in this study and exposure history data so far, the improvement of surveillance system that can link them is required to estimate an accurate *R*_0_. Second, the number of available sequences in the database is limited. There would be difference in the sampling probability between human and avian sequences. Assuming that the sampling probability in human sequences is higher than that of avian sequences, we may have overestimated the *R*_0_. Third, we did not model movement of hosts, both of human and avian due to the lack of data. Modeling movement of hosts explicitly can improve accuracy of estimates. Fourth, our method can applicable only for infections in sporadic outbreaks and cannot be used for infections in an endemic situation, since the length of transmission chain is assumed follow the geometric distribution.

The small value of *R*_0_ estimate, i.e., below unity, suggests that human-to-human transmission of H5N1 viruses in Egypt is limited. The large increase in human cases in Egypt since 2014 is likely attributed to other factors than the increase in human-to-human transmission potential. Therefore the increase in human cases should be attributed to the increase in avian-to-human transmissions. A possible cause of the increase in avian-to-human transmissions is the spread of the viruses in avian populations, which increase the chance of avian-to-human transmissions. Another possibility is the increase in the susceptibility of humans to infection with these H5N1 viruses due to virus evolution. Thus, monitoring the spread and evolution of viruses in avian population as well as those in human populations is required to prevent major outbreaks of H5N1 infections among the human population.

## Data Availability Statement

All datasets generated for this study are included in the article/Supplementary Material.

## Author Contributions

WM and RO designed the study. WM and KI analyzed the data. WM, KI, and RO wrote the manuscript.

## Conflict of Interest

The authors declare that the research was conducted in the absence of any commercial or financial relationships that could be construed as a potential conflict of interest.

## Supplementary Material

TABLE S1 The GenBank accession numbers, strain names, sampling dates, and sampling locations of nucleotide sequences used in this study.

TABLE S2 Sensitivity analysis of *R*_0_ against genetic distance, time difference, geographical distance. Lb and ub are the upper and lower bound of 95% confidence interval for the *R*_0_ estimate.

DATA SHEET S1 R codes for estimation of *R*_0_ and its confidence interval from phylogeny, genetic distance, time difference, and geographical distance.

DATA SHEET S2 Detailed process of statistical analysis for estimation of *R*_0_ and its confidence interval from phylogeny, genetic distance, time difference, and geographical distance.
