# Supplementary material for: Estimating Transmission Potential of H5N1 Viruses Among Humans in Egypt Using Phylogeny, Genetic Distance and Sampling Time Interval
Source: Front Microbiol. 2019 Dec 3;10:2765. doi: 10.3389/fmicb.2019.02765 (PMC6901801; doi:10.3389/fmicb.2019.02765)
Supplement: DATA SHEET S2 — Detailed process of statistical analysis for estimation of R0 and its confidence interval from phylogeny, genetic distance, time difference, and geographical distance. [file Data_Sheet_2.DOCX]

**Supplemental Text**

**Estimating Transmission Potential of H5N1 Viruses among Humans in Egypt Using Phylogeny, Genetic Distance and Sampling Time Interval**

**Wessam Mohamed^1^, Kimihito Ito^1^, Ryosuke Omori^1^***

^1^Division of Bioinformatics, Research Center for Zoonosis Control,

Hokkaido University, Sapporo, Japan

*** Correspondence:**

Ryosuke Omori

omori@czc.hokudai.ac.jp

# Collection of the human isolate clusters in the phylogeny

The genetic distance among avian isolates and human isolates were visualized using a phylogenetic tree. If two human isolates are connecting with one or two internal nodes in the phylogenetic tree, they are considered to belong to the same cluster (human 1 and human2; human3 and human4 in Figure S1a and S1b). If two human isolates are connecting with more than two internal nodes in the phylogenetic tree, they are considered to belong to different clusters (human5 and human6 in Figure S1c). Applying this criterion for all pairs of human isolates, we get clusters of human isolates. A cluster with a single human isolate is called a singleton cluster.

Figure S1. Human isolate clusters in the phylogeny

For each cluster containing more than one human isolates, human isolates were sorted in ascending order according to their sampling dates. Accessions of two human isolates adjacent each other in the sorted order were recorded as candidate transmission pairs. Sampling time interval and geographical distance between candidate transmission pairs were recorded. The list candidate transmission pairs can be found in the candidate_pairs_from_phylogeny.csv.

# Estimation of *R*_0_ with phylogeny

For each candidate transmission pair found using phylogeny, we tested whether the pair satisfy the criteria of genetic distance, sampling time interval, and geographical distance. Pairs satisfying criteria is considered as human-to-human transmission. *R*_0_ was estimated using the formula $R_{0}=(S-n)/S$ where $S$ is total number of human sequences (=60) and $n$ is the number of avian to human transmissions. Here, $n$ is equal to $S$ minus the number of human-to-human transmissions, and $(S-n)$ is equal to the number of human-to-human transmissions. The 95% confidence intervals are calculated by the profile likelihood method, which finds$\theta$ satisfying $2\left( \log\left( L\left( \hat{\theta};x_{1},\ldots,x_{n} \right) \right)-\log\left( L\left( \theta;x_{1},\ldots,x_{n} \right) \right) \right)\leq3.84$, where $\hat{\theta}$ is estimated *R*_0_. The R code for estimating *R*_0_ and its 95%CI can be found in estimate_R0_phylogeny.r. The sensitivity analyses were conducted by running the code by changing thresholds values of interest.

# Estimation of *R*_0_ without phylogeny

From genetic distance, sampling time interval, and geographical distance among human isolates, we collected clusters of human isolates. Two human isolates were considered to belong the same cluster if they satisfy the threshold criteria on genetic distance, sampling time interval, and geographical distance. A human isolate that did not satisfy the threshold criteria with another human isolates were considered as a singleton cluster. The DBSCAN algorithm (Ester et al., 1996) was implemented and used to find clusters. The clustered sequences were sorted by their sampling time, and two human isolates adjacent each other in the sorted order were considered as candidate human-to-human transmission pairs. *R*_0_ was estimated using the formula $R_{0}=(S-n)/S$ where $S$ is total number of human sequences (=60) and $n$ is the number clusters found by the DBSCAN algorithm. The 95% confidence intervals are calculated by the profile likelihood method, which we have described in the precious section. The supplementary file estimate_R0_no_phylogeny.r contains the R code for clustering and the estimation *R*_0_ and its 95%CI. The sensitivity analyses were conducted by running the code by changing thresholds values of interest.

**Reference**

Ester, M., Kriegel, H.P., Sander, J., and Xu, X. (1996). "A density-based algorithm for discovering clusters in large spatial databases with noise", in: *Proceedings of the Second International Conference on Knowledge Discovery and Data Mining (KDD-96)*. AAAI Press.
